# Adherence and long-term growth outcomes: results from the easypod^™^ connect observational study (ECOS) in paediatric patients with growth disorders

**DOI:** 10.1530/EC-18-0172

**Published:** 2018-07-05

**Authors:** Ekaterina Koledova, George Stoyanov, Leroy Ovbude, Peter S W Davies

**Affiliations:** 1 Biopharma Global Medical AffairsGeneral Medicine and Endocrinology, Merck KGaA, Darmstadt, Germany; 2 Business & Decision Life SciencesBrussels, Woluwe-Saint-Lambert, Belgium; 3 Children’s Nutrition Research CentreFaculty of Medicine, University of Queensland, Brisbane, Queensland, Australia

**Keywords:** adherence, easypod, GH treatment, e-Health

## Abstract

**Objective:**

The easypod connect observational study (ECOS) assessed treatment adherence among paediatric patients receiving growth hormone (GH) via the easypod electronic injection device.

**Design:**

ECOS was an open-label, observational, longitudinal study conducted in 24 countries between 2010 and 2016, enrolling children treated with GH.

**Methods:**

The primary endpoint was the rate of treatment adherence during 5 years of follow-up. Impact of adherence on growth outcomes was assessed using Spearman’s product–moment correlations.

**Results and conclusions:**

Overall, 1190 patients had easypod data available for ≥3 months; most patients had GH deficiency (75%); 606 of these patients were GH naïve at baseline. Over the first year of monitoring, the median rate of adherence was 93.7% among patients overall and >93.0% in GH-naïve patients, irrespective of the treatment indication. Clinically meaningful improvements in growth rates were observed after 1 year of treatment across all GH indications. Adherence decreased with increasing treatment duration, but the overall median adherence rate remained high after 3 years of follow-up: 87.2% (*n* = 409), 75.5% after 4 years (*n* = 143) and 70.2% after 5 years (*n* = 43). Statistically significant correlations between adherence and 1-year change in height standard deviation score (*P* < 0.001 for patients overall) and height velocity (*P* < 0.001) were observed.

**Conclusions:**

ECOS produced accurate, real-time adherence data in a large population of GH-treated children over 5 years of follow-up. Using the easypod connect system, physicians can potentially identify patients with inadequate adherence and poor response to treatment, enabling them to take appropriate action to help them maximise the benefits of GH treatment.

## Introduction

Recombinant human growth hormone (GH) is approved for use in the treatment of children with various aetiologies, including growth hormone deficiency (GHD), Turner syndrome (TS) and born small for gestational age (SGA) with no catch-up growth (1, 2, 3). The aim of GH treatment is to initiate catch-up growth and improve adult height and metabolic parameters, which requires long-term commitment from the patient and/or carer for regular injections. The length of continuous prior GH treatment (in GH-naïve or non-naïve patients) is known to be a major factor contributing to the response to GH treatment (4, 5). However, motivation may decrease over time because the benefits of GH treatment are not immediately apparent, and daily subcutaneous injections may present a significant burden (6). Poor adherence to the injection regimen is a major concern in the management of growth disorders, and regular assessment of adherence is an essential component of successful treatment (6, 7, 8). Studies have indicated that poor adherence to GH therapy is common and is associated with decreased efficacy outcomes and increased healthcare costs (7, 9, 10, 11).

Detection of poor adherence to GH therapy can be difficult because patients or carers may be reluctant to admit to it and may overestimate reported rates (12). Even theoretically objective measures, such as checking diary cards or the number of prescriptions collected, do not reliably confirm that doses have been taken (6). Nevertheless, early recognition of non-adherence is essential in the identification and subsequent prevention of technical, physical and/or psychological barriers to adherence. A number of factors have been identified to help healthcare professionals and patients maintain or improve adherence to treatment with GH (11). Improved ease and perceived convenience of administering treatment can have a positive impact on the level of adherence, leading to favourable growth outcomes (7, 12).

The easypod device is currently the only electronic GH injection device available; it is approved for use in more than 40 countries so far, including the European Union and the United States. It was awarded the gold medal at the 2007 Medical Design Excellence Awards (13) and received the 2017 Pharmaceutical Market Excellence Awards innovation for e-Health award (14). Easypod was developed to improve patient convenience during long-term GH treatment (15) and to provide accurate, unbiased data on an individual patient’s adherence to treatment by real-time recording of the timing, date and dose of GH delivered (12, 16, 17). The patient shares these data with their healthcare provider at intervals, using the easypod connect web-based software, which stores the data in a secure database. For patients with chronic diseases, their caregivers and healthcare providers, digital tools are becoming important for enabling improved management of the long-term outcomes of the condition (18). Electronic monitoring with easypod may help patients and healthcare providers to open a dialogue around the benefits of adherence and work together to maintain this aspect of treatment (8). The easypod technology may be particularly helpful for patients who tend to forget doses but are unaware of the number actually missed and has been described as a novel example of triage between patient, carer and healthcare professional (11). In particular, it enables physicians to closely monitor adherence rates and outcomes and intervene as appropriate.

The easypod connect observational study (ECOS) was a 5-year investigation to assess treatment adherence among paediatric patients receiving GH via the easypod device. Secondary objectives included investigation of any correlation between adherence, socioeconomic factors and changes in long-term growth outcomes.

## Materials and methods

### Patients and study design

ECOS was an open-label, observational, longitudinal study conducted in 24 countries (Argentina, Australia, Austria, Canada, China, Colombia, Czech Republic, Finland, France, Greece, Hungary, Indonesia, Italy, Kingdom of Saudi Arabia, Korea, Mexico, Norway, Singapore, Slovakia, Spain, Sweden, Taiwan, United Arab Emirates, United Kingdom) between November 2010 and February 2016. Adherence data in Spain were not collected via easypod connect and, therefore, Spanish results were not included in this analysis; interim results for Spain have been published separately (19).

Patients included in the study were aged 2–18 years or >18 years without fusion of growth plates and were receiving GH via the easypod device (Saizen^®^, Merck KGaA, Darmstadt, Germany). Eligible patients from each of the participating countries were enrolled in the database and attended one baseline visit followed by 1–4 visits per year, according to local routine clinical practice. Planned duration of follow-up was at least every 6 months for up to 5 years. Owing to the observational nature of the study, all diagnoses and treatment decisions were at the discretion of the investigating physician, following standard endocrine practice.

The study was conducted in accordance with the principles of the Declaration of Helsinki, Good Clinical Practice (ICH-GCP E6) guidelines and applicable national legal and regulatory requirements. Written informed consent was obtained from patients (or their parent/guardian) prior to study enrolment. The study was approved by local ethical committees for the centres in each country and the details for these may be found in the online Supplementary data (see section on supplementary data given at the end of this article).

### Data collection and study endpoints

Adherence data were primarily derived from injections recorded in the easypod device, while baseline and outcome measures were obtained by physician data entry into clinical report forms. A protocol amendment was made after the start of the study to capture GH-naïve status. GH naïve status in a patient was defined as any patient who had not been treated with any GH formulation before starting treatment with easypod.

The primary endpoint was the rate of treatment adherence derived from the easypod device, defined as the percentage of prescribed injections that were recorded as being administered. Variables assessed with respect to adherence in this study included prior exposure to GH preparations, the indication for GH treatment, age at easypod start (<6 years or ≥6 years; younger children were expected to have had their injections conducted under parental supervision and to have shown better growth response to therapy than older children (16, 20, 21)), gender, Tanner stage at easypod start (1 and >1) and parental sociodemographic factors (marital and employment status). Secondary endpoints included changes from baseline in growth outcomes and insulin-like growth factor 1 (IGF1) concentrations.

Suspected serious adverse events (SAEs) that occurred during the study were collected and reported directly to the sponsor’s Global Drug Safety Officer. Adverse events were coded according to the current Medical Dictionary for Regulatory Authorities (MedDRA) version in the sponsor’s Global Safety Database (ARIS) at the time of SAE reporting (22).

### Statistical analysis

This population-based observational study aimed to generate hypotheses relating to influencers of adherence and, therefore, was not limited in terms of sample size. The primary analysis population was the easypod adherence data analysis set (DAS), comprising patients who had a recorded treatment start date, no gaps >1 week of recorded injections, height measurements available at both treatment start and 1 year (±3 months) and who had at least 3 months of data from the easypod device. Data were analysed for these patients overall, and for the subgroup of GH-naïve patients.

Adherence rates were calculated on a cumulative basis, by duration of easypod use and for individual treatment periods (from the beginning of treatment to the last complete week with adherence data available for each patient). Because of the single-arm, observational nature of the study, the statistical analysis was descriptive, with summary statistics for primary and secondary endpoints. Height standard deviation score (SDS) was calculated using World Health Organization reference data (23) and height velocity (HV) SDS was calculated using Tanner growth standards (24). The impact of adherence rates on clinical outcomes at the end of 1 year of treatment was analysed using Spearman’s product–moment correlations. Two previously published definitions of clinically relevant response to GH treatment were used, as derived from studies of GH-naïve patients: change in height SDS ≥0.5 (more stringent) and HV SDS ≥+1 (less stringent) (25).

## Results

### Patients

A total of 2420 patients were enrolled in ECOS, of whom 1203 had sufficient data for inclusion in the study, and 1190 patients had easypod data for ≥3 months and comprised the easypod adherence DAS; 606 of these patients were GH naïve at baseline. The main indication for GH treatment was GHD (75% of patients); the remaining patients had SGA (17%), TS (7%) or ‘other’ (1%; including chronic renal failure/chronic kidney disease, short stature/slow growth and unspecified indication). A summary of baseline demographic characteristics and auxological data is presented in Table 1.

**Table 1:** Baseline patient demographic and auxological data for patients treated with GH using easypod, overall and for the subgroup who were GH naïve at the start of easypod use.

	Overall analysis population (*n* = 1203)	GH-naïve population (*n* = 610)
Age, years (min; max)	10 (1; 19)	10 (1; 19)
Sex, *n* (%)		
Female	503 (41.8)	238 (39.0)
Male	700 (58.2)	372 (61.0)
Ethnicity, *n* (missing)	1185 (18)	593 (17)
African, *n* (%)	9 (0.7)	4 (0.7)
Asian, *n* (%)	195 (16.2)	108 (17.7)
Caucasian, *n* (%)	822 (68.3)	367 (60.2)
Other, *n* (%)	159 (13.2)	114 (18.7)
Missing	18 (1.5)	17 (2.8)
Pubertal stage, *n* (missing)	490 (713)	573 (37)
Tanner 1, *n* (%)	335 (68.3)	416 (72.6)
Tanner >1, *n* (%)	155 (21.7)	157 (27.4)
IGF1 status, *n* (missing)	390 (813)	109 (501)
Abnormal low, *n* (%)	51 (13.1)	13 (11.9)
Normal, *n* (%)	300 (76.9)	88 (80.7)
Abnormal high, *n* (%)	39 (10.0)	8 (7.3)
Bone age, *n* (missing)	207 (996)	507 (103)
Greulich and Pyle assessment (years)	10.0 (6.0; 12.0)	8.0 (5.0; 10.6)
Growth velocity (cm/year)	4.6 (3.3; 5.1)	4.0 (3.3; 5.1)
Height (cm)	121.1 (103.2; 134.4)	122.8 (106.3; 135.0)
Indication for GH treatment, *n* (%)		
GHD	897 (74.6)	499 (81.8)
SGA	207 (17.2)	67 (11.0)
Turner syndrome	82 (6.8)	37 (6.1)
Other	17 (1.4)	3 (0.5)

Unless specified, values are presented as median (Q1; Q3) or number of patients (% of total).

CAS, complete analysis set; GH, recombinant human growth hormone; GHD, growth hormone deficiency; IGF1, insulin-like growth factor-1; SGA, small for gestational age.

### Adherence

Adherence data were available for at least 1 year for >98% of patients in the easypod adherence DAS. Over the first year of monitoring, the median rate of adherence was 93.7% among patients in both the easypod adherence DAS and the subgroup of GH-naïve patients. Median adherence rates were >93.0% in patients in each of the indication groups, including GHD (93.4%), SGA (95.0%) and TS (93.2%). In the easypod adherence GH-naïve subgroup, adherence was >90.0% for patients with GHD, irrespective of the cause: idiopathic isolated GHD (92.3%), organic GHD of congenital origin (91.1%) or tumour origin (96.4%).

**Figure 1:**
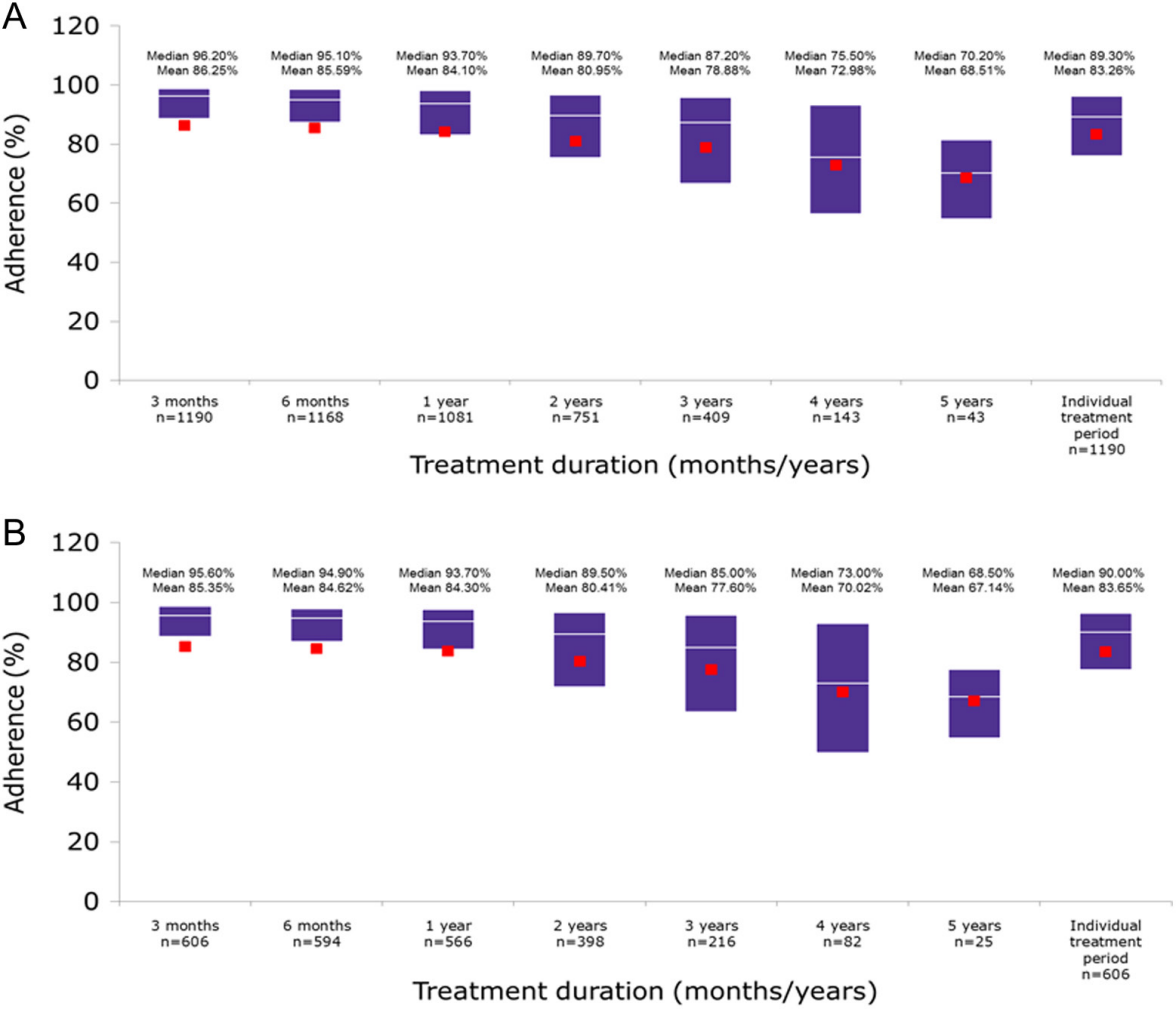
Treatment adherence rates over time in the easypod adherence data analysis set (A) overall (*n* = 1190) and (B) the GH-naïve patients (*n* = 608). Boxes show Q1 and Q3, with median as white line and mean as red squares.

**Figure 2:**
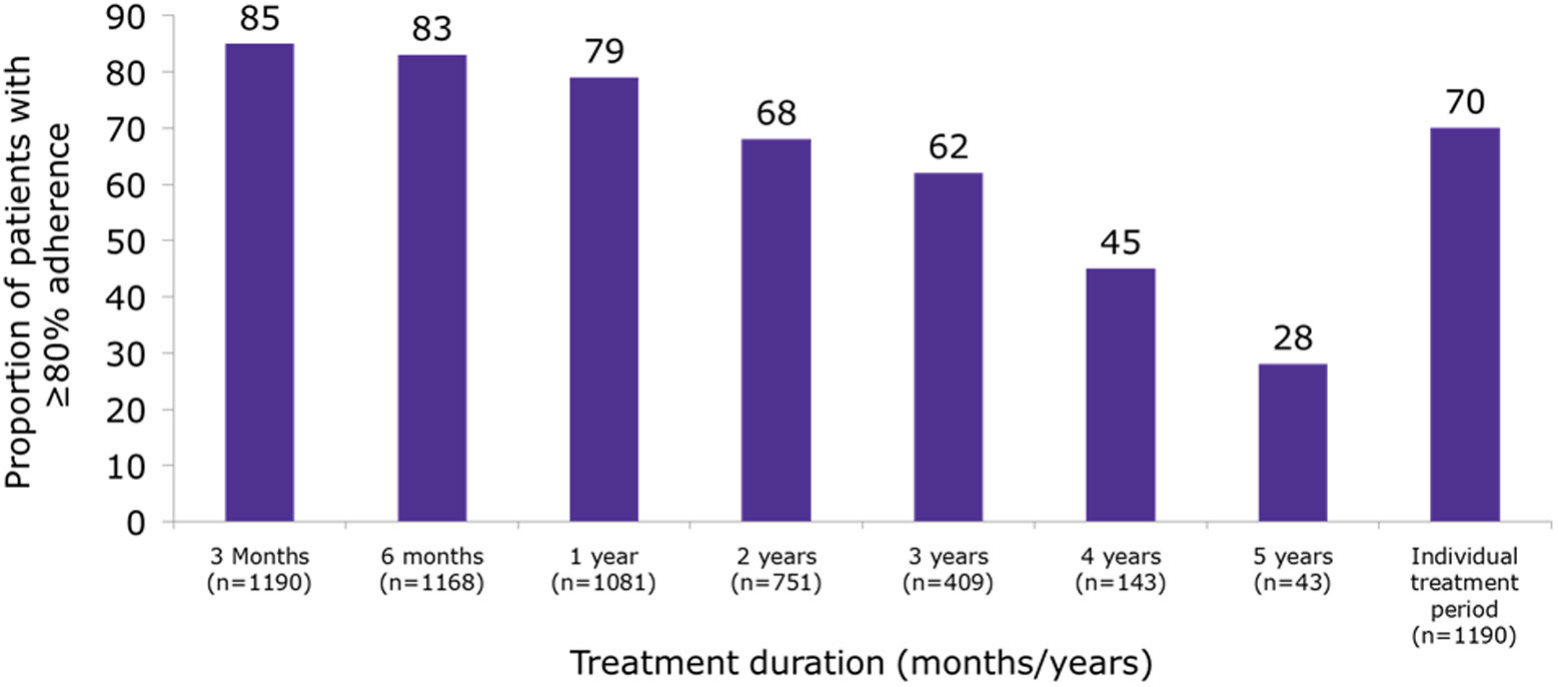
The proportion of patients treated with GH using ‘easypod’ with adherence rates of at least 80% at each year over the 5-year study period and for all patients at any time within the 5-year period.

Although adherence decreased with increasing duration of use, median adherence rate in the easypod adherence DAS (Fig. 1) was still high: 87.2% after 3 years of follow-up (*n* = 409), 75.5% after 4 years of follow-up (*n* = 143) and 70.2% after 5 years of follow-up (*n* = 43). The proportion of patients with an adherence rate of ≥80% (Fig. 2) was 79.0% after 1 year, 68% after 2 years, 62% after 3 years, 45% after 4 years and 28% after 5 years. Assessment of individual patient’s treatment periods showed 70.0% of patients had adherence rates of ≥80%. Similar adherence trends were observed in the GH-naïve subgroup. In the easypod adherence DAS, adherence trends were similar over 5 years for the indications of known origins.

### Growth outcomes

Within the easypod adherence DAS, overall, the median change in height SDS from baseline to 1 year was 0.47 (Q1; Q3 0.27; 0.68) across each of the indication groups; median overall HV over the first year of treatment was 8.2 (6.9; 9.4) cm/year and HV SDS was 2.11 (0.60; 3.62), indicating a positive growth response (Table 2A). The growth response in patients with TS was the lowest among the three main indications (as assessed by change in height SDS and HV SDS).

**Table 2:** Growth outcomes and changes from baseline after 1 year of GH treatment using easypod for patients (A) overall and (B) who were GH naïve at start of easypod use.

Growth outcome	GHD (*n* = 886)	SGA (*n* = 206)	Turner syndrome (*n* = 82)	Other^a^/missing (*n* = 16)	Overall (*n* = 1190)
**(A) Easypod adherence data analysis set**					
Baseline height SDS (s.d.)	−2.15 (0.98)	−2.45 (0.95)	−2.47 (0.96)	−2.41 (0.80)	−2.23 (0.98)
Change in height SDS at 1 year	0.47 (0.27; 0.69)	0.50 (0.29; 0.68)	0.41 (0.19; 0.65)	0.44 (0.15; 0.51)	0.47 (0.27; 0.68)
Baseline height velocity (cm/year)	4.00 (3.20; 5.00)	4.84 (3.92; 5.60)	4.08 (3.21; 5.55)	4.10 (3.60; 4.68)	4.00 (3.30; 5.10)
1-year height velocity (cm/year)	8.2 (6.9; 9.5)	8.3 (7.1; 9.3)	7.2 (5.6; 8.7)	8.0 (6.2; 8.8)	8.2 (6.9; 9.4)
1-year height velocity SDS	2.20 (0.62; 3.83)	2.09 (0.73; 3.3)	1.38 (−0.10; 2.19)	1.63 (0.80; 3.13)	2.11 (0.60; 3.62)
**Growth outcome**	**Idiopathic isolated GHD** (*n* = 166)	**Organic GHD** (congenital) (*n* = 23)	**Organic GHD** (tumour) (*n* = 27)	**Non-GHDb** (*n* = 390)	**Overall** (*n* = 606)
**(B) Easypod adherence data analysis set – GH-naïve**					
Baseline height SDS (s.d.)	−2.27 (0.95)	−3.15 (1.15)	−1.53 (1.22)	−2.25 (0.88)	−2.26 (0.95)
Change in height SDS at 1 year	0.55 (0.35; 0.73)	0.72 (0.35; 1.03)	0.37 (0.15; 0.74)	0.47 (0.27; 0.69)	0.50 (0.28; 0.72)
Baseline height velocity (cm/year)	4.00 (3.50; 5.00)	4.33 (3.27; 7.00)	2.90 (1.70; 6.00)	4.00 (3.30; 5.08)	4.00 (3.30; 5.08)
1-year height velocity (cm/year)	8.4 (7.2; 9.7)	8.7 (7.3; 10.5)	8.20 (6.4; 9.3)	8.2 (6.9; 9.5)	8.3 (7.1; 9.6)
1-year height velocity SDS	2.54 (1.39; 3.65)	2.63 (0.67; 4.44)	2.35 (0.83; 4.18)	2.04 (0.59; 3.62)	2.15 (0.83; 3.69)

Values are presented as mean (s.d.) or median (Q1; Q3) unless stated otherwise.

^a^Other indications include chronic renal failure/chronic kidney disease, short stature/slow growth and other; ^b^non-GHD includes patients with missing GHD origin.

GH, growth hormone; GHD, growth hormone deficiency; s.d., standard deviation; SDS, standard deviation score; SGA, small for gestational age.

Among GH-naïve patients, the greatest change in height SDS was observed in those with organic GHD of congenital origin (median change in height SDS 0.72 (0.35; 1.03) and HV SDS 2.63 (0.67; 4.44)) followed by idiopathic isolated GHD (median change in height SDS 0.55 (0.35; 0.73) and HV SDS 2.54 (1.39; 3.65); Table 2B).

### Correlation of adherence with growth outcomes

Spearman’s product–moment correlations between adherence rate and growth measures (Table 3A) were 0.11 (*P* < 0.001) for change in height, 0.13 (*P* < 0.001) for change in height SDS, 0.14 (*P* < 0.001) for HV and 0.08 (*P* = 0.013) for HV SDS, indicating a positive correlation between adherence rate and growth response. Positive correlation values were also generally observed in each of the indication groups, although these only reached statistical significance for the GHD patients.

**Table 3:** Correlation of adherence with growth outcomes after 1 year of GH treatment using easypod for patients (A) overall and (B) who were GH naïve at start of easypod use.

Growth outcome	GHD (*n* = 886)	SGA (*n* = 206)	Turner syndrome (*n* = 82)	Other^a^/missing (*n* = 16)	Overall (*n* = 1190)
**(A) Easypod adherence data analysis set**					
Change in height, *n* (missing)	803 (83)	188 (18)	76 (6)	14 (2)	1081 (109)
Spearman’s product–moment correlation	0.12	0.08	0.11	−0.22	0.11
*P* value	<0.001	0.283	0.329	0.454	<0.001
Change in height SDS, *n* (missing)	803 (83)	188 (18)	76 (6)	14 (2)	1081 (109)
Spearman’s product–moment correlation	0.14	0.13	0.05	−0.32	0.13
*P* value	<0.001	0.0870	0.6692	0.2668	<0.001
Height velocity, *n* (missing)	803 (83)	188 (18)	76 (6)	14 (2)	1081 (109)
Spearman’s product–moment correlation	0.13	0.17	0.17	−0.09	0.14
*P* value	<0.001	0.0196	0.1491	0.7708	<0.001
Height velocity SDS, *n* (missing)	790 (96)	187 (19)	68 (14)	14 (2)	1059 (131)
Spearman’s product–moment correlation	0.11	−0.06	0.03	−0.39	0.08
*P* value	0.002	0.4294	0.8121	0.1745	0.013
**Growth outcome**	**Idiopathic isolated GHD** (*n* = 167)	**Organic GHD** (congenital) (*n* = 23)	**Organic GHD** (tumour) (*n* = 27)	**Unspecified/non-GHDb** (*n* = 391)	**Overall** (*n* = 608)
**(B) Easypod adherence data analysis set – GH-naïve**					
Change in height, *n* (missing)	150 (16)	19 (4)	23 (4)	374 (16)	566 (40)
Spearman’s product–moment correlation	−0.04	0.28	−0.32	0.13	0.07
*P* value	0.627	0.255	0.139	0.011	0.110
Change in height SDS, *n* (missing)	150 (16)	19 (4)	23 (4)	373 (17)	565 (41)
Spearman’s product–moment correlation	0.02	0.18	−0.26	0.16	0.09
*P* Value	0.794	0.457	0.236	0.002	0.025
Height velocity, *n* (missing)	150 (16)	19 (4)	23 (4)	373 (17)	565 (41)
Spearman’s product–moment correlation	0.04	0.29	−0.34	0.15	0.09
*P* value	0.632	0.237	0.114	0.005	0.028
Height velocity SDS, *n* (missing)	147 (19)	18 (5)	23 (4)	362 (28)	550 (56)
Spearman’s product–moment correlation	0.06	0.18	−0.29	0.10	0.06
*P* value	0.464	0.483	0.175	0.055	0.133

The *P* values provided were not adjusted for multiplicity of testing.

^a^Other indications include chronic renal failure/chronic kidney disease, short stature/slow growth and other; ^b^non-GHD included patients with GHD of unspecified origin in addition to non-GHD indications.

GH, growth hormone; GHD, growth hormone deficiency; SDS, standard deviation score; SGA, small for gestational age.

Equivalent growth outcome and correlation data were also obtained from the GH-naïve subgroup (Table 3B). Growth outcome data generally indicated a positive growth response, greater than that observed for patients in the easypod adherence DAS. Among the indication groupings presented, patients with organic GHD of congenital origin generally achieved the highest growth response, followed by patients with idiopathic isolated GHD. Patients with organic GHD of tumour origin achieved lower median growth responses than those in the other GHD groups.

For each indication, positive correlations were seen in the easypod adherence DAS for patients with GHD, which constituted the majority of the assessed population: the Spearman’s product–moment values were 0.14 (*P* < 0.001) and 0.11 (*P* < 0.01) for the correlations between adherence rate and change in height SDS and adherence rate and HV SDS, respectively. With regard to the GH-naïve subgroup, Spearman’s product–moment correlations between adherence rate and measures of growth response were generally slightly lower than those for the overall group and consistently failed to reach statistical significance across the different measures.

### Insulin-like growth factor-1 concentrations

In the adherence DAS, IGF1 concentrations after 1 year were within the normal range in the majority of patients, and only a small proportion of patients had either abnormally elevated or abnormally low concentrations. No SAEs due to abnormally high IGF1 concentrations were reported. However, IGF1 concentrations at 1 year were not available for 51.8% of patients, so no correlation analysis with adherence was performed.

### Safety

For patients included in the easypod adherence DAS (*n* = 1190), the overall mean (±s.d.) duration of treatment was 935.2 (±456.5) days, with a mean GH dose of 0.03 (±0.01) mg/kg/day administered at study entry (data available for *n* = 1185). In the GH-naïve subgroup (*n* = 606), the overall mean duration of treatment was 964.6 (±464.5) days and mean dose at study start was 0.03 (±0.02) mg/kg/day.

A total of 75 SAEs were reported in 53 patients during this study. Twelve SAEs (16%) in 11 patients were considered to be related to Saizen (Table 4) and 63 SAEs (84%) were not related to study drug and were confounded by the subject’s medical history, concurrent conditions and/or concomitant use of medications. In seven of the 53 cases, treatment with the study medication was discontinued due to the event and was not re-introduced. One death occurred during the study. A 15-year-old male with a history of granulomatous disease experienced intermittent fever, several episodes of non-bloody vomiting, milky discharge from abdominal fistula, pneumonia, large pneumothorax and clotting around the peripherally inserted central catheter line. He died approximately 1.5 months after stopping GH treatment. All events except pneumonia had resolved prior to the subject’s death. The death was reported as related to treatment by the mother, but as not related by the investigator, and no further information was available.

**Table 4:** Serious adverse events considered related to study medication.

MedRA Preferred Term	Number of events
Acute lymphocytic leukaemia	1
Adenoidal disorder	1
Blood thyroid stimulating hormone increased	1
Death	1
Gynaecomastia	1
Headache	1
Hyperglycaemia	1
Neoplasm recurrence	1
Nephrotic syndrome	1
Scoliosis	1
Sleep apnoea syndrome	1
Tympanic membrane disorder	1
IGF1 status, n (missing)	1
**Total**	**12**

## Discussion

The global 5-year ECOS is the first large-scale study to provide an objective assessment of adherence and effects on growth outcomes during GH treatment with the easypod device in paediatric patients with growth disorders. Median adherence rates were high (94%) over the first year of study treatment, and only gradually decreased with longer duration of follow-up. An adherence rate of ≥80% was maintained by the majority of patients over 3 years of treatment and over each individual treatment period. The associations between high adherence rates and positive growth outcomes were statistically significant in the overall adherence DAS population.

The easypod device has been associated with good adherence among paediatric patients requiring GH treatment in previous shorter-term observational studies (12, 16, 17). In a 3-month study (*n* = 824), the recorded dose history showed adherence of 87.5%, with significantly higher adherence in treatment-naïve patients than in treatment-experienced patients (12). In another study (*n* = 75), a median adherence rate of 96.0% was recorded over a period of 343 days (16). In a 12-month study of 97 prepubertal patients, high levels of adherence were recorded, with 57% of patients having adherence rates of ≥92% (17); treatment was associated with improved height, and IGF1 levels were within the normal range in the majority of patients. A single-centre study involving 23 patients who switched to easypod, generally because of concerns about prior adherence, showed median adherence to treatment of 99% over a 6-month period, while median adherence in a cohort from the same centre using pen devices was 82% (21).

In the present study, which includes both GH treatment-naïve and non-naïve patients, clinically meaningful improvements in growth rates were observed after 1 year of treatment across all GH indications, based on a HV SDS cut-off of ≥1. Using the more stringent change in height cut-off of ≥0.5 SDS, only patients in the GH-naïve subgroup with idiopathic isolated GHD and organic GHD of congenital origin had growth rates considered clinically meaningful (25). Although a decrease was seen over time, median adherence was still high at >75% after 4 years. Similar results were observed for the indications of GHD, SGA and TS, which suggests that adherence was not strongly influenced by underlying diagnosis. However, the decreased adherence over time suggests the need for structured and active interventions from healthcare practitioners, patient support programmes and carers to manage adherence over the course of GH treatment (18). The most common reasons for missed injections were ‘forgot injections’ (70.3%) and holidays/long weekends (36.2%). Active interventions to manage adherence were not a part of this study, owing to its observational design. Different adherence enhancement techniques may have been applied (e.g., cognitive motivation, alerts (18, 19)), but data regarding such interventions were not collected. Further studies are required to address this underexplored area of patient care.

Among patients in the GH-naïve subgroup, those with organic GHD of tumour origin maintained the highest adherence rates over time, despite lower growth responses, particularly compared with patients with idiopathic isolated GHD. This might reflect good recognition by this group of the importance of treatment adherence in order to maintain the effects of GH beyond catch-up growth. As expected, patients in the GH-naïve subgroup with GHD of congenital origin showed the highest growth rates.

Generally, positive correlations between adherence and growth outcomes were observed overall. These trends support an interpretation that adherence is a necessary contributor to an adequate clinical response to GH treatment. When assessed by diagnosis, statistical significance was only reached for patients with GHD, although this could be due to the small number of subjects with available data in the other groups; it should also be noted that statistical analyses were not adjusted for multiplicity of testing and should be interpreted with caution. Positive correlations between adherence rate and the 1-year changes in height and HV were seen in the GHD group, supporting the clinical relevance of monitoring adherence. Among GH-naïve patients, Spearman’s product–moment correlations between adherence and growth outcomes after 1 year were not significant. However, this may be due to the very high rates of adherence observed in the first year of treatment, and possibly also to the smaller numbers of patients involved. Different statistical methods with longer than 1 year follow-up can provide additional insights to these correlations.

Limited analyses of sociodemographic data suggested that patients with a married or cohabiting parent had higher median adherence rates after 1 year of easypod use than those with a separated or divorced parent (6% and 5% between-group differences in adherence rates for marital status of mothers or fathers, respectively). There were no clear differences in adherence rates according to age (<6 years or ≥6 years), gender, pubertal stage or parents’ employment status. Further investigations of potential confounders are needed to more accurately quantify the observed trends.

The limitations of this study include its non-interventional nature, which is associated with a high level of missing data, high inter-patient variability, inclusion of both GH treatment-naïve and non-naïve patients and the absence of detailed recording of actions performed by healthcare providers and carers when poor adherence and/or poor response to treatment was recorded. However, these limitations occur in all surveillance studies (26), whereas the observational nature means that it reflects normal clinical practice. Strengths include that it was the first study that used a device with an e-Health platform to report adherence data directly from patients to healthcare providers, its prospective design, the 5-year duration and the large number of patients enrolled and followed up. A number of individual cases from ECOS have been reported (19, 27, 28, 29); these indicate that direct access to adherence monitoring can make the difference in a patient’s management and motivation. Examples include cases of suboptimal adherence when pubertal catch-up growth was observed, better understanding of complex cases with poor response to treatment despite excellent adherence, and identification of risk factors for poor adherence (27, 28, 29).

No other large-scale patient registries have provided comparable insights into patient adherence to GH treatment; this is because of the lack of devices comparable to easypod for enabling assessment of real-time adherence (30). Other smaller scale studies have assessed adherence to GH treatment, but most have relied on either questionnaire-based reporting by patients/carers (31, 32, 33, 34, 35), vial/cartridge accountability (9) or prescription refill rate (6, 20, 21). Furthermore, these studies, and some larger multicentre studies, have provided only limited detail concerning adherence trends over time. Similar to the present study, some reports have indicated a trend towards decreasing adherence over time; however, the magnitude of these changes cannot be objectively assessed. A study in Turkey (31) that focused exclusively on GH-naïve patients using pen devices for GH administration revealed a progressive decline in reported adherence over the first year of treatment; this finding is in contrast with ECOS, in which adherence over the first year was high and sustained.

## Conclusions

ECOS has provided accurate, robust and real-time adherence data in a large population of patients receiving GH via easypod. The study showed that through using easypod and easypod connect, physicians can identify patients with inadequate adherence, which will enable them to take appropriate action to help maximise the benefits of GH treatment. Poor adherence to treatment is an issue for the management of most chronic diseases, and early detection to enable improved adherence in patients receiving GH is crucial to achieve normal adult height. The study also confirmed the value of using an e-Health platform to monitor adherence to obtain better outcomes. Statistically significant associations were found between adherence and growth outcomes, supporting the monitoring of adherence. No new safety signal was identified in ECOS, and the benefit–risk balance for GH via easypod remains favourable.

## Supplementary Material

Supporting Table 1

## Declaration of interest

E K and G S are employees of Merck KGaA, Darmstadt, Germany. P S W D and L O have received honoraria and research grants from Merck KGaA, Darmstadt, Germany.

## Funding

This study was sponsored by Merck KGaA, Darmstadt, Germany. Medical writing assistance was provided by David Candlish, inScience Communications, Tattenhall, UK, and sponsored by Merck KGaA, Darmstadt, Germany.

## Author contribution statement

E K, G S, L O and P S W D contributed to the data analysis, review and interpretation and writing/revisions/approval of drafts and final manuscript. L O also provided biostatistical services.
