# Supplementary material for: Adherence and long-term growth outcomes: results from the easypod™ connect observational study (ECOS) in paediatric patients with growth disorders
Source: Endocr Connect. 2018 Jul 5;7(8):914–23. doi: 10.1530/EC-18-0172 (PMC6107763; doi:10.1530/EC-18-0172)
Supplement: Supporting Table 1 [file ec-7-914-t001.pdf]

Easypod™ Connect Observational Study (ECOS) local ethical committees

Country	Site Number	Date of Protocol Approval	Local Ethical Committee
**Argentina**	171	06 June 2012	Comité independiente de ética para Ensayos en Farmacologia Clínica,
	359	11 February 2014	Comité independiente de ética para Ensayos en Farmacologia Clínica
**Australia**	020	24 December 2010	Maters Health Services Human Research Ethics Committee
	021	14 February 2011	Austin Health Human Research Ethics Committee
	022	1 February 2011	ACT Health Human Research Ethics Committee.
	025	28 March 2011	Monash Health Human Research Ethics Committee
	036	24 May 2011	Princess Margaret Hospital for Children Ethics Committee
**Austria**	018/023/026	21 December 2010	Single approval for all sites by EC.<br>EC of the medical University Vienna and of the general hospital of the city Vienna<br>AKH Borschkegasse 8b / E06<br>A-1090 Vienna
**Canada**	024	06 April 2011	UBC C&W Research Ethics Board
	017	06 January 2011	University of Western, Office of Research Ethics Board HSREB
	008	07 January 2011	Comite d’ethique du CHU Sainte-Justine
	185	09 May 2012	Service de soutien à l'éthique de la recherche en santé sur l'humain Centre hospitalier universitaire de Sherbrooke
	120	24 October 2011	Health Research Ethics Board
	010	13 June 2011	SickKids – Research Ethics Board
	287	22 March 2013	Health Research Ethics Board – University of Manitoba
	009	18 November 2010	Conjoint Health Research Ethics Board – University of Calgary
	272	30 April 2012	Horizon Health Network – Research Ethics Board
	019	25 February 2011	Hamilton lntegrated Research Ethics Board – McMaster University
**China**	071	13 September 2011	EC of the first affiliated hospital,Sun Yat-sen University
	072	09 August 2011	EC of Beijing Children´s Hospital,capital medical university
	073	09 August 2011	Ethics Committee of Peking union medical college hospital
	074	13 October 2011	Ethics Committee of Chengdu Women & Children´s central Hospital
	103	12 October 2011	Ethics Committee of Fujian

			province,Fuzhou Children´s Hospital
	104	30 November 2011	Ethics Committee of Sun Yat-sen Memorial Hospital Sun Yat-sen University
	105	03 November 2011	Ethics Committee of Children´s Hospital of Fudan University
**Columbia**	361	20 November 2013	Hospital San Jose Ethics Committee
	362	30 April 2014	Fundación Cardio Infantil Ethics Committees
**Czech Republic**	050	29 June 2011	Etická komise FN Motol<br>V Úvalu 84, Praha 5, 150 06
	048	10 June 2011	Etická komise, Krajská zdravotní, a.s. - Masarykova nemocnice v Ústí nad Labem, o.z.<br>Sociální péče 3316/12A,<br>401 13 Ústí nad Labem
	047	11 July 2011	Etická komise FN a LF UP Olomouc<br>I. P. Pavlova 185/6<br>779 00 Olomouc
	049	23 June 2011	Etická komise Endokrinologického ústavu<br>Národní 8, 116 94 Praha 1
	292	09 February 2012	Etická komise FN Plzeň<br>E. Beneše 13, 305 99 Plzeň
	046	24 August 2011	Etická komise FN Brno<br>Jihlavská 20, 625 00 Brno
	067	17 December 2012	Etická komise FN Hradec Králové<br>Sokolská 581, 500 05 Hradec Králové
	143	01 February 2012	Etická komise Fakultní nemocnice Královské Vinohrady<br>Šrobárova 1150/50, Praha 10, 100 34
**Finland**	331	21 October 2013	Helsingin Ja Uudenmaan (local EC at site level is not applicable – Central Ethics Approval
**France**			CCTIRS (Comité Consultatif sur le Traitement de l'Information en Matière de Recherche dans le Domaine de la Santé).
**Greece**	085	02 February 2011	Local institutional review board of Athens University Medical School
	249	28 April 2011	Local institutional review board of “P.&A. Kyriakou” Children’s Hospital
	252	28 April 2011	Local institutional review board of “P.&A. Kyriakou” Children’s Hospital
**Hungary**	144, 145, 146, 151, 152, 156, 176, 177, 204, 275	19 January 2012	Central Ethics - Egészségügyi Tudományos Tanács Tudományos és Kutatásetikai Bizottsága, ETT TUKEB (Scientific and Research Ethics Committee of the Medical Research Council)
**Indonesia**	371	22 Apriol 2013	Health Research Ethics Committee Faculty of Medicine University of Indonesia and Cipto Mangunkusumo

			Hospital.
**Italy**	091	19 October 2011	Comitato Etico della ASL di Cagliari
	140	19 January 2012	Comitato Etico dell'Università Cattolica del Sacro Cuore - Policlinico Universitario Agostino Gemelli di Roma
	092	14 December 2011	Comitato Etico dell'Azienda Ospedaliera Santobono-Pausilipon di Napoli
	207	21 December 2011	Comitato Etico dell'Azienda Ospedaliera Policlinico Consorziale di Bari
	138	21 December 2011	Comitato Etico della ASL LE di Lecce
	093	29 November 2011	Comitato di Etica dell'IRCCS Istituto Giannina Gaslini di Genova
	180	10 January 2012	Comitato Etico dell'IRCCS Centro di Riferimento Oncologico di Basilicata di Rionero in Vulture (PZ)
	164	27 January 2012	Comitato Etico dell'Azienda Ospedaliera Ospedali Riuniti Villa Sofia-Cervello di Palermo
	345	11 January 2012	Comitato Etico per le Attività Biomediche Carlo Romano dell'Università degli Studi Federico II di Napoli
	128	22 February 2012	Comitato Etico per la Sperimentazione Clinica dei Medicinali dell'Azienda Ospedaliera Universitaria Integrata di Verona
	181	12 March	Comitato Etico Per la Sperimentazione dell'Azienda Ospedaliera di Padova
	183	24 January 2012	Comitato Etico Unico per la Provincia di Parma
	119	15 December 2011	Comitato Etico dell'IRCCS Ospedale Casa Sollievo della Sofferenza di S. Giovanni Rotondo (FG)
	127	19 December 2011	Comitato Etico per la Sperimentazione Clinica dell IRCCS Ospedale Pediatrico Bambino Gesù di Roma
	141	23 January 2012	Comitato Etico Scientifico dell'Azienda Ospedaliera Universitaria Policlinico Gaetano Martino di Messina
	184	22 March 2012	Comitato Etico della ASL 4 di Teramo
	187	26 April 2012	Comitato Etico Sperimentazione Clinica Medicinali della AUSL 8 di Arezzo

	356	17 December 2013	Comitato Etico dell'Azienda Ospedaliero-Universitaria Ospedali Riuniti Umberto I-G.M. Lancisi-G.Salesi di Ancona
	322	17 December 2013	Comitato Etico per la Sperimentazione dei Farmaci dell' Azienda Ospedaliero-Universitaria A. Meyer di Firenze
	327	19 June 2013	Comitato Etico dell'Azienda Ospedaliera Policlinico Consorziale di Bari
	333	26 June 2013	Comitato Etico Locale per la Sperimentazione Clinica dell' Azienda Ospedaliera L. Sacco di Milano
	325	08 July 2013	Comitato Etico dell`Azienda Ospedaliera Universitaria Policlinico Paolo Giaccone dell`Università degli Studi di Palermo
	332	27 June 2013	Comitato Etico dell'IRCCS Istituto Auxologico Italiano di Milano
**Kingdom of Saudi Arabia**	368	16 March 2014	Institutional Review Board University of Dammam PO Box 2114 Dammam 31451 KSA
	351	07 August 2014	Institutional Review Board KFMC P.O. Box 59046 Riyadh 11525 Kingdom of Saudi Arabia
**Korea**	015	30 November 2010	Severance Hospital Yonsei university health system Institutional Review Board
	016	21 October 2010	Institutional Review Board of Ajou University Hospital
	040	28 July 2011	Chonnam National University Hospital Institutional Review Board
	041	13 June 2011	Institutional Review Board of Inje university Pusan Paik hospital
	042	28 June 2011	Asan Medical Center Institutional Review Board
	045	08 August 2011	Korea University Anam Hospital Clinical Trial Center Institutional Review Board
	043	10 June 2011	Kyungpook national University Hospital Institutional Review Board
	070	07 November 2011	The Catholic University of Korea Seoul ST. Mary's Hospital Institutional Review Board
	044	27 July 2011	Chungbuk national university hospital Institutional Review Board
**Mexico**	131,	06 March 2012	Comite Bioetico para la lnvestigacion Clinica S.C.
	132, 133, 134, 135, 136, 165, 166	14 February 2012	Comite Bioetico para la lnvestigacion Clinica S.C.

**Norway**	All sites	12 May 2014	The Regional Committees for Medical and Health Research Ethics (REK) – Central Ethics Approval
**Singapore**	375	17 June 2014	SingHealth Centralised Institutional Review Board
	376	23 May 2014	NHG Domain specific review board
**Slovakia**	All sites	14 September	Etická komisia DFNsPCentral Ethics Approval
**Spain**	190	4 July 2011	Hospital General Universitario Gregorio Marañón Ethics Board
	120000985	24 April 2013	Hospital Universitario De Girona Ethics Board
**Sweden**	028	24 October 2010	EPN - Regionala Etikprövningsnämnden i Stockholm – Central Ethics Approval
**Taiwan**	343	26 November 2013	Institutional Review Board of Kaohsiung Medical University Hospital
	344	25 December 2013	Taipei Medical University Joint Institutional Review Board
**United Arab Emirates**	349	01 April 2014	Ethics and Research Committee Mafraq Hospital
**United Kingdom**	All sites		Ethics approval not required
